# Granulate-to-Filament: An Extrusion-Mixed PLA–Human Bone Material System for 3D-Printed Bone Scaffolds

**DOI:** 10.3390/jfb17040187

**Published:** 2026-04-11

**Authors:** Jonas Neijhoft, Hela Weslati, Volker Eras, Jan Brune, Maximilian Leiblein, Santiago Bianconi, Nicolas Söhling, Lewin Busse, René Verboket, Johannes Frank, Ingo Marzi, Dirk Henrich

**Affiliations:** 1 Department of Trauma and Orthopaedics, Goethe University Frankfurt, Jonas Neijhoft, Theodor-Stern-Kai 7, 60528 Frankfurt am Main, Germany; 2 German Institute for Cell and Tissue Replacement gGmbH (DIZG), 12555 Berlin, Germany

**Keywords:** bone tissue engineering, long bone defects, additive manufacturing, immunomodulation, multifunctional scaffolds, 3D-printing diamond concept, electrostimulation, bio-functionalization

## Abstract

Fused filament fabrication (FFF) enables patient-specific scaffolds for critical-size bone defects, but most filaments are bioinert and difficult to functionalize at high particulate loadings due to segregation, agglomeration, clogging, and diameter instability. We developed a mechanism-guided extrusion toolkit to stabilize polylactic acid (PLA) filaments containing human demineralized bone matrix (DBM) or cortical granulate (CG) up to 70 wt%. PLA was ground, dried, silicone pre-coated, and compounded with DBM or CG (25/40/70 wt%) using starve-fed extrusion, sequential extrusion, and post-die mixing to maintain stable diameters. FFF produced disks and tubes. MSC adhesion was assessed by SEM. qPCR (control vs. osteogenic medium) quantified RUNX2, ALP, BGLAP, COL1A, VEGF, IL-6, MAPK8. Tubes underwent three-point bending. The toolkit yielded printable, dimensionally stable filaments at 25–70 wt% with uniform dispersion and surface-exposed filler. Both composites increased early mesenchymal stromal cells (MSC) adhesion versus PLA. RUNX2 was increased on DBM40 versus PLA. VEGF was elevated on CG25 (DBM40 trend). Under osteogenic medium, IL-6 and MAPK8 were generally reduced. Mechanics were loading-dependent: CG25 exceeded CG70 and DBM25, while DBM40/70 recovered stiffness versus DBM25. A mechanism-guided extrusion toolkit enables high-loading PLA–DBM/CG filaments with excellent printability and material-specific biological and mechanical advantages over PLA.

## 1. Introduction

Critical-size bone defects (CSDs)—defects that will not heal spontaneously—remain a major clinical challenge in orthopedics and trauma surgery, driving substantial morbidity and cost [1].

Standard reconstructions (e.g., autograft, allograft) are constrained by limited supply, donor-site morbidity, and variable integration, motivating biomaterial strategies to reliably bridge these defects [1,2]. Nevertheless, any bone substitute must be measured against this gold standard of iliac-crest cancellous autograft. To be truly effective, it should reproduce the core elements of the diamond concept by providing osteoconductive architecture, osteoinductive cues, osteogenic cells, rapid vascularization, and sufficient mechanical stability [3].

In the German population, fracture-related (aseptic) non-unions show an incidence of 17.4/10,000 in 2019 [4], and the presence of a non-union economically increases the treatment costs up to 2.6 to 4.3 times compared to uncomplicated fractures [5,6]. In the US, the treatment costs range from $33,000 to $85,000 per case, depending on the variability of the necessity of re-operations [7]. Furthermore, due to the long treatment time, it adds fundamental costs regarding productivity losses [8]. Beyond the clinical complexity, this shows the socio-economic impact, which is estimated to be around $142,000 per case in Germany [6]. Additive manufacturing enables the fabrication of patient-specific scaffolds with controlled porosity, architecture, and mechanical properties. Among available printing modalities, fused filament fabrication (FFF) is attractive for translational applications because of its accessibility, scalability, and ability to produce reproducible constructs with defined macro- and microarchitectures [9,10,11,12,13,14]. Based on numerous studies, including our own, polylactic acid (PLA) is a widely adopted FFF polymer due to its processability and cytocompatibility [15,16,17]. However, as a synthetic aliphatic polyester, it exhibits limited intrinsic bioactivity (manifested by low cell adhesion and osteogenic signaling) and relatively slow, acid-generating hydrolytic degradation, both of which may impair direct osseointegration unless bioactive functionality is introduced [18,19]. To evaluate the biological potential of such functionalized surfaces, mesenchymal stromal cells (MSCs) are commonly employed as a predictive in vitro model and are essential for bone healing due to their osteogenic differentiation potential. Since their early attachment and signaling response are highly sensitive to substrate properties, they serve as a critical biological indicator to validate whether the introduced bioactivity effectively compensates for the inert nature of PLA [20,21,22,23].

Two clinically relevant bioactive components to address this gap are demineralized bone matrix (DBM) and cortical granulate (CG). DBM supplies endogenous osteoinductive proteins, predominantly collagen type I, within an osteoconductive matrix and has a long history of safe use as a graft extender or substitute in bone repair [24,25]. However, owing to its proteinaceous and collagen-dominated composition, DBM rapidly loses shape and structural integrity in aqueous environments, limiting its standalone handling and load-bearing capability. Despite these limitations, DBM has demonstrated favorable healing outcomes in several studies performed by our group [25,26,27].

CG, the granulated mineral fraction of cortical bone with embedded proteins, is highly biocompatible and osteoconductive but lacks osteoinductive properties. Therefore, when used alone, CG typically requires reinforcement or composing to achieve both mechanical stability and biological performance. Furthermore, most of these filling materials are available in a granular form, which inherently lacks mechanical integrity and stability. [25,28].

A composite approach—embedding bioactive fillers in a printable PLA matrix—has therefore emerged as a pragmatic path to combine structural integrity with biological performance, and several studies already document improved osteogenic and even pro-angiogenic responses when PLA is combined with bioactive phases, such as bioactive glass (BG), validating the concept for bone repair [1,25,29]. Additionally, these bioactive fillers are intended to enhance osteoconductivity by the scaffolds themselves and to trigger osteoinductive signaling pathways through the release of endogenous growth factors (e.g., BMPs). Furthermore, the integration of such particulates allows for a modulation of the mechanical profile.

However, incorporating high loadings of biologically active, often irregular or hydrated particulates (such as DBM or CG-based additives) into thermoplastic filaments while maintaining printability and diameter stability remains technically challenging. Processing strategies adapted from polymer engineering—e.g., starve-fed extrusion and sequential/masterbatch compounding to improve dispersion—can mitigate agglomeration and thermal history in composite filaments, thereby enabling higher additive contents without compromising filament quality [30,31,32].

Against this backdrop, the present work focuses on the manufacturing of bioactive composite filaments using DBM and CG incorporated within a PLA matrix. We describe a practical, mechanism-informed process chain (granulation, silicone coating, starved feeding/sequential extrusion, and post-extrusion mixing) to achieve printable, diameter-stable filaments at clinically relevant high filler loadings. Their printability and in vitro performance were benchmarked by comparing PLA, PLA–DBM, and PLA–CG filaments. This manufacturing-first perspective aims to bridge the gap between benchtop formulation and robust, 3D-printable, bioactive filaments suitable for preclinical translation.

## 2. Materials and Methods

### 2.1. PLA Feedstock Preparation

Polylactic acid (PLA, Ingeo Biopolymer PLA, Natureworks, Plymouth, MN, USA) in the form of commercial granulates (nominal granule size 1–3 mm) was used as the polymer matrix and as the pure PLA control. In a first preparation step, the raw PLA was ground to obtain a finer feed size of approximately 0.25–1 mm using a shredder (QITech Industries GmbH, Darmstadt, Germany). The resulting PLA granulate was then dried at 40 °C for 24 h to reduce moisture prior to processing. No additives or plasticizers were introduced at any stage. Following drying, the material was stored closed until use.

### 2.2. Blend Preparation

To ensure stable extrusion, especially when small volume amounts of biological filler are added, a minimum batch size of 10 g was defined for each formulation. To ensure a homogenous distribution and prevent agglomeration, the PLA–DBM and PLA–CG composite granules were prepared using a multi-step coating process. Briefly, raw PLA granules were placed in a 50 ml Falcon tube and pre-coated with three drops (~60–90 mg) of silicone oil per 10 g (J.Harhaus, Radevormwald, Germany). The granules were vortexed to distribute the microfilm evenly over all pellet surfaces. The pre-coated PLA was then transferred into a fresh Falcon tube to avoid wall-retained residues from the initial container and to start blending on a clean surface. Under continuous vortexing, the calculated amount of DBM or CG (DIZG, Berlin, Germany) was incrementally added until the target wt% was reached. The PLA control group underwent identical silicone pre-coating and vortexing protocol (three drops, same tubes and handling).

By this silicone-assisted pre-coating, a uniform distribution can be promoted, and segregation during feeding and extrusion was suppressed. To ensure that the hopper remains filled for a continuous flow during preloading and that the low overall quantity of 10 g can be maintained, a small amount (100 mg) of black filament is added towards the end of the process. As soon as this appeared at the extruder tip, the process was stopped. This then serves as an end marker for the processed filament and guarantees sufficient and consistent filler dosage.

The masterbatch of—in this case—PLA–DBM(CG) composite granules is then used for the extrusion of filament as described in the following section.

### 2.3. Filament Extrusion and Consistency Assessment

Composite granules were processed under starved feed extrusion, which can be described as controlled under-feeding to increase specific shear and promote PLA wetting and deagglomeration of DBM or CG without over-pressurizing the composite.

For very high loadings with up to 90%, a masterbatch pass was followed by pelletization and a dilution pass to the final wt%, improving dispersion by breaking weak filler–filler contacts formed on cooling. Immediately downstream of the die, the semi-molten strand passed a short post-extrusion mixing section to damp residual concentration waves before sizing and spooling. Extrusion was performed at 4.4 rpm with a four-zone temperature profile: T1 175 °C, T2 186 °C, T3 188 °C, T4 171 °C. Active cooling fabs were operated at 100%.

Filament diameter was monitored with a target of 1.75 ± 0.1 mm. All formulations (PLA, PLA–DBM, PLA–CG at 25/40/70 wt%) produced dimensionally stable, FFF-capable filaments. 

To assess filler dispersion and filament consistency, cross-sections of PLA, PLA–DBM, and PLA–CG filaments (25/40/70 wt%) were examined by scanning electron microscopy (SEM) as described in the SEM section. Sections were prepared orthogonally to the draw direction and imaged to document the spatial distribution of particulate phases within the PLA matrix and the presence of voids or streaking.

### 2.4. Fabrication of Test Specimens by FFF

**Figure 1:**
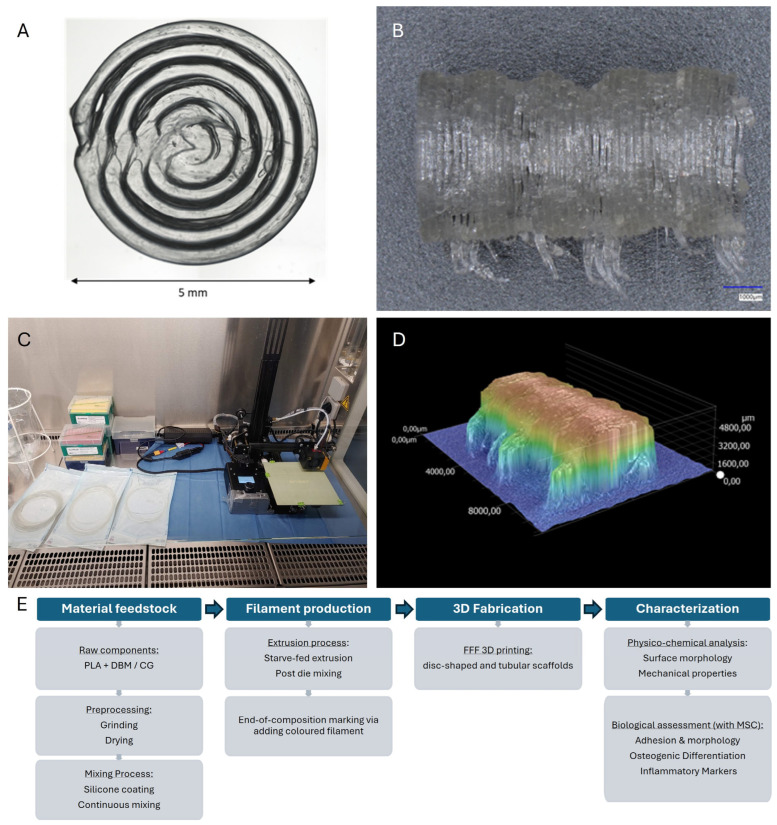
Printed test specimens and workflow overview. Geometry and architecture of FFF-printed test specimens. (**A**) Two-layer disk (Ø = 5 mm, layer height = 0.2 mm) used for cell adhesion, morphology, and gene expression studies. The surface pattern is hypothesized to provide a beneficial microtopography for cell attachment. (**B**,**D**) Cylindrical three-dimensional scaffold (outer Ø = 5 mm, inner lumen Ø = 4.2 mm, length = 6 mm, wall thickness = 0.4 mm) for three-point bending tests, shown in (**B**) perspective view and (**D**) topographical surface view. Both geometries were printed from PLA, PLA–DBM, and PLA–CG filaments at 0, 25, 40, and 70 wt% filler loadings on a Voron 2.4 and under sterile conditions (**C**) on a modified Ender-2 desktop printer (nozzle temperature 185 °C, bed temperature 25 °C, 0.4 mm nozzle). (**E**) Systematic flow chart of the experimental pipeline, detailing the stages from feedstock preparation and the specialized extrusion toolkit to final physico-chemical and biological characterization.

Filaments were processed by FFF into disk-shaped and tubular test specimens for biological and mechanical assessments, respectively. Printing was performed using standard PLA processing parameters with a nozzle temperature of 185 °C and bed temperature of 25 °C. Printing parameters were kept constant across materials within each experiment to enable direct comparisons of biological and mechanical readouts and to demonstrate the printability of filaments with high additive loadings, which tend to be more complex to print. To assess robustness and transferability, specimens were fabricated not only on a high-end CoreXY printer (Voron 2.4, Afterburner Revo Hotend, 0.4 mm nozzle size), but also on a modified low-cost Ender-2 (Creality, Shenzhen, China), featuring a direct-drive extruder modification with a 0.4 mm nozzle size. Printing was conducted under sterile conditions. Test specimens were not further sterilized for the test groups, as the inherent sterility of the printing process has been demonstrated previously [33]. Representative geometries of the printed test specimen are shown in Figure 1.

### 2.5. Cell Culture and Experimental Conditions

In vitro experiments were performed using a pooled population of human mesenchymal stromal cells (MSCs) derived from five independent donors, as previously described by Söhling et al. (2022). [19]. The isolated hMSCs exhibited a typical mesenchymal stromal cell phenotype (CD73^+^, CD90^+^, CD105^+^, CD45^−^, CD34^−^) and demonstrated trilineage differentiation potential. The human samples used in this study were provided by the German Red Cross (DRK) Blood Donor Service, Baden-Württemberg–Hessen, and were approved for experimental purposes by the Ethics Committee of the Department of Medicine, Goethe University (vote no. 329/10). 

MSCs were cultured under standard conditions (37 °C, 5% CO_2_, humidified atmosphere) in animal-free growth medium (GM) consisting of 5% heparin-free PLTGold^®^ human platelet lysate (hPL; Merck KGaA, Darmstadt, Germany), 100 U/mL penicillin, and 100 µg/mL streptomycin (Sigma-Aldrich, St. Louis, MO, USA) in GlutaMAX™ DMEM low glucose (Gibco, Thermo Fisher Scientific, Dreieich, Germany). The culture medium was changed three times per week, and cells were passaged at approximately 80% confluence. For passaging, cells were detached using 0.05% trypsin–EDTA (Gibco, Thermo Fisher Scientific, Dreieich, Germany). MSCs at passage 5 were used for all experiments.

For cell adhesion and surface topology analyses, an amount of 10,000 MSCs was seeded onto test specimens (constructs) and assessed after 24 h of culture. For osteogenic differentiation experiments, constructs were cultured either in GM or in osteogenic differentiation medium consisting of GM supplemented with 10 mM β-glycerophosphate disodium salt hydrate, 50 µM ascorbic acid 2-phosphate, and 10 nM DEXA (Sigma-Aldrich, St. Louis, MO, USA) and analyzed at days 7 and 14.

### 2.6. Cell Adhesion and Surface Topology

Cell attachment was assessed on disk-shaped specimens, as described in our previous studies [27]. To prevent specimen flotation in culture medium, 10 µL hPL was pipetted into the designated wells of a 24-well plate. After 15 min, test specimens were gently placed onto the residual hPL droplet, which effectively anchored each specimen to the well bottom. For cell seeding, 1 × 10^4^ MSC (passage 5) suspended in 15 µL of GM were carefully deposited centrally onto each specimen. The seeding volume was chosen to allow uniform spreading of the droplet across the entire surface without disruption due to surface tension. After an initial adhesion period of 30 min at 37 °C in a humidified atmosphere with 5% CO_2_, additional GM was carefully added, and the specimens were incubated for 24 h.

Cell attachment on the specimen was quantified using Trypan Blue staining. After rinsing to remove non-adherent cells, disks were incubated in Trypan Blue solution (5 min). Shortly, for each disk, the culture medium was completely aspirated, and the surface was washed twice with sterile PBS. To contextualize cell adhesion results, surface topography was imaged using a digital microscope (BZ-X810, Keyence, Osaka, Japan) with coaxial and oblique illumination and optical depth-stacking. The Trypan Blue-positive surface area was measured using ImageJ software (version v1.54d, National Institutes of Health, Bethesda, MD, USA) and expressed as a fraction of the total specimen surface [34].

#### Scanning Electron Microscopy (SEM)

Disk-shaped test specimens without cells and seeded with MSCs (10,000 cells) were processed for SEM to visualize early homogeneity, cell adhesion, and morphology after 24 h. Samples were fixed in 2% glutaraldehyde in PBS without Ca^2+^ and Mg^2+^ for 10 min, and subsequently dehydrated through a graded ethanol series (50%, 75%, 96%, 100%; 5 min each). Following dehydration, specimens were briefly transferred to 1,1,1,3,3,3-hexamethyldisilazane (HMDS) (Merck Schuchardt, Hohenbrunn, Germany), allowed to drain overnight, and sputter-coated with gold (5 × 60 s) using an Agar Sputter Coater (Agar Scientific Ltd., Stansted, UK). SEM imaging was performed using a Hitachi scanning electron microscope (Hitachi, Düsseldorf, Germany), and micrographs were acquired and handled with Digital Image Processing System 2.6 (Point Electronic, Halle, Germany). All sample preparation steps and imaging parameters were kept consistent across material groups and time points.

### 2.7. Gene Expression of Osteogenic, Inflammatory, and Pro-Angiogenic Markers 

After removal of the culture medium, disk-shaped specimens were washed twice with sterile Ca^2+^/Mg^2+^-free PBS. Total RNA was extracted by adding RLT lysis buffer (Qiagen, Hilden, Germany) directly to the adherent cells, followed by purification using the RNeasy Mini Kit (Qiagen, Hilden, Germany) according to the manufacturer’s instructions. RNA concentration and purity (A260/A280 ratio) were determined using a NanoQuant plate in a spectrophotometer (Infinite 200 Pro, Tecan, Grödig, Austria). Complementary DNA (cDNA) was synthesized from DNase-treated RNA using the iScript cDNA Synthesis Kit (Bio-Rad, Hercules, CA, USA). Quantitative PCR was performed using iTaq Universal SYBR Green Supermix (Bio-Rad, Hercules, CA, USA) with a cDNA input corresponding to 8 ng of RNA per reaction. Amplification was carried out on a CFX96 Touch Real-Time PCR Detection System (Bio-Rad, Hercules, CA, USA) using commercially available human gene-specific primers (Qiagen, Hilden, Germany). Melting curve analysis was conducted to confirm amplification specificity. 

Assessment was performed at day 7 and day 14 from cells on PLA, PLA–DBM, and PLA–CG (25/40/70 wt%) under control and osteogenic (OD) media. Gene expression levels were normalized to GAPDH, and relative expression was calculated using ΔCt and fold-change, with the corresponding negative control group (PLA control at each time point/medium) used as the calibrator.

### 2.8. Mechanical Testing

Tubular and untreated test specimens from each filament composition were evaluated by a three-point bending test using a tabletop material testing machine (Zwickiline 5.0, Zwick-Roell, Ulm, Germany). Prior to testing, specimens were conditioned at room temperature. During testing, load was recorded while the plunger was lowered at a constant speed of 0.1 mm/s. The breaking load was defined by a force drop of at least 50%. Breaking loads and bending stiffness were calculated using the software testXpert II (Zwick Roell, Ulm, Germany). N = 6 specimens per group were tested. An overview of the whole workflow can be seen in Figure 1E.

### 2.9. Statistics

Results are displayed as boxplots centered on the median (boxes indicate the interquartile range, whiskers extend according to the Tukey method unless stated otherwise). Group comparisons across more than two categories were conducted using the nonparametric Kruskal–Wallis test followed by Dunn post hoc analyses. Pairwise comparisons (e.g., two time points, such as day 7 vs. day 14, or two groups at a matched condition) were performed using the Wilcoxon–Mann–Whitney U test. Dichotomous data were analyzed using Fisher’s exact test. All statistical tests were two-sided, with α = 0.05 considered statistically significant. Statistical analyses and figure generation were performed using Bias 11.12 (Epsilon-Verlag, Darmstadt, Germany) and GraphPad Prism Ver. 10.2 (GraphPad Software, Boston, MA, USA).

## 3. Results

### 3.1. Filament Blending, Feed Consistency, and 3D-Printing

**Figure 2:**
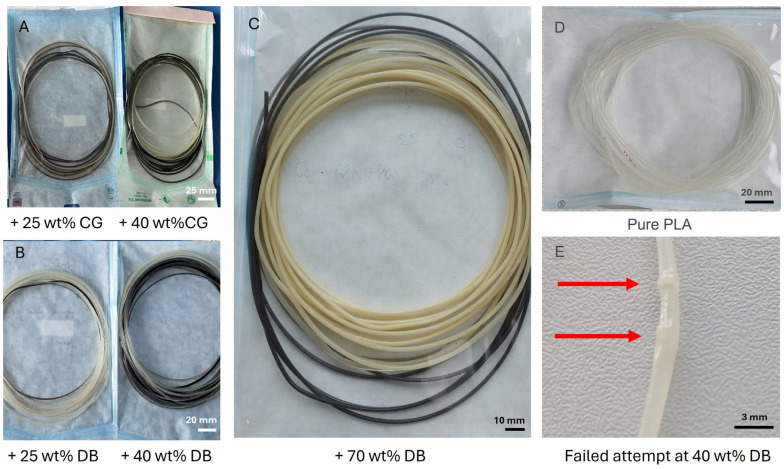
Filament appearance and processing consistency. Visual appearance of PLA–CG (**A**,**C**) and PLA–DBM (**B**) composite filaments across increasing filler loadings (exemplary shown for 25, 40, and 70 wt%) and pure PLA filament (**D**). All filaments were extruded to a target diameter of 1.75 ± 0.1 mm and then sterile-packed in bags and stored at 4 °C until use. The black segment visible at the end of each spool is a process-end marker and is left on the spool for easier filament insertion into the extruder. A progressive increase in opacity with rising CG or DBM content is evident. In contrast, initial trials prior to process optimization resulted in inhomogeneous filaments (**E**), characterized by particle agglomerations and diameter fluctuations (red arrows). These defects were particularly visible at higher filler loadings and resulted in unprintable filaments when not using the extrusion optimization procedures. Scale bars and reference scale in the lower corner (**A**)= 25 mm, (**B**) = 20 mm, (**C**) = 10 mm, (**D**) = 20 mm, (**E**) = 3 mm).

Composite filaments containing CG or DBM were successfully fabricated at filler contents of 25, 40, and 70 wt% and could be spooled at a controlled diameter suitable for FFF. All formulations were reproducibly printable, enabling the fabrication of 2D disk-shaped specimens for cell culture assays as well as 3D porous cylindrical constructs for mechanical testing. Macroscopic inspection of printed specimens revealed increasing opacity and appearance changes with rising DBM content, consistent with the increasing mineral or matrix load within the polymer phase. To ensure that the hopper remains filled with continuous flow during preloading and that the low overall quantity of 10 g can be maintained, a small amount (50 mg) of black filament is added towards the end of the process. As soon as this appears at the extruder tip, the process can be stopped. This then serves as an end marker for the processed filament and guarantees sufficient and consistent filler dosage (Figure 2). The optimization process to overcome the filament defects shown in Figure 2E primarily focused on the described procedures, such as homogeneous filler loadings by silicone distribution and starve feeding, and the synchronization of screw speed and pull-off velocity. Initial trials with standard extrusion settings resulted in diameter fluctuations and surface pitting due to the high particulate loading (Figure 2E). After optimization, a more uniform particle distribution and stabilized filament diameter of 1.75 ± 0.1 mm could be achieved (Figure 2C).

SEM of filament cross-sections demonstrated a homogeneous distribution of both CG and DBM within the PLA matrix across all concentrations examined. The distribution suggests that particles located near the periphery contribute to an increased surface irregularity, which likely facilitates the enhanced MSC adhesion observed in the biological assays. While these cross-sections primarily show the internal architecture, the protrusion of granules at the edges provides a qualitative indication of the modified surface topography compared to the smooth profile of pure PLA.

**Figure 3:**
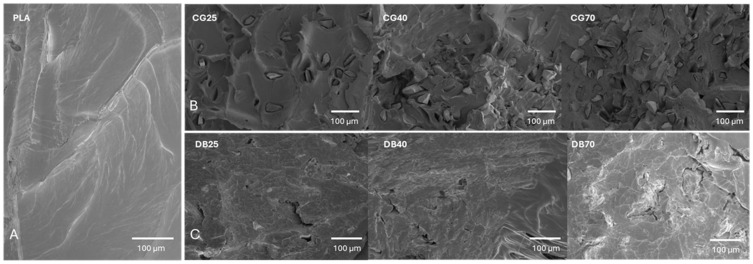
Scanning electron micrographs (SEM) of filament fracture surfaces (cross-sections). Pure PLA (**A**) reference showing a smooth, homogeneous polymer matrix with characteristic brittle fracture lines. (**B**) PLA–CG composites at 25, 40, and 70 wt% loadings. CG appears as distinct, angular inclusions embedded within the polymer matrix. The particle density increases progressively with loading, yet the particles remain well-distributed without large agglomerates. (**C**) PLA–DBM composites at 25, 40, and 70 wt% loadings. DBM particles exhibit a more fibrous, textured morphology integrated into the PLA. Note the consistent dispersion across all concentrations, confirming the efficacy of the manufacturing process (granulation/coating/starved feeding) in preventing phase separation. CG = CG, DB = DBM.

In printed disk-shaped specimens, the filler particles were partially exposed on the surface in a concentration-dependent manner. These exposed inclusions generated localized topography features with lateral dimensions of ~5–10 µm in width and up to ~50 µm in length. Surface roughness increased with higher filler fractions and showed different appearances for CG and DBM: whereas DBM, with its fibrous structure, is more embedded into the filament, CG gives a rougher texture (Figure 3).

### 3.2. Biomechanics

Mechanical characterization by three-point bending tests of the tubular specimens revealed distinct, composition-dependent trends. Within the CG series, CG25 displayed a significantly higher bending stiffness compared with CG70 and DB25, and with higher values than pure PLA. 

Within the DBM series, DB25 showed a slight decrease in bending stiffness compared with PLA, while DB40 and DB70 exhibited similar increases in stiffness, both being significantly higher than DB25. Direct cross-series comparison showed that CG25 was significantly stiffer than DB25.

**Figure 4:**
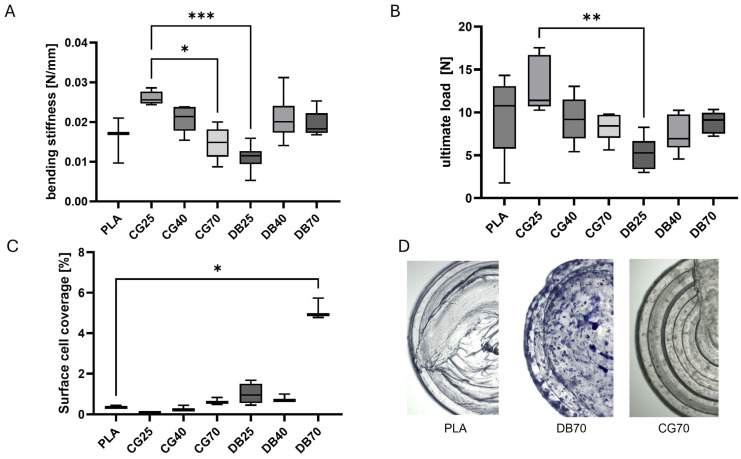
Mechanical properties and cell adhesion on 3D-printed composite scaffolds. (**A**,**B**) Three-point bending analysis of cylindrical test specimens. (**A**) Bending stiffness (N/mm) and (**B**) ultimate load (N) of PLA and composite scaffolds with increasing CG or DBM content. Data are displayed as box plots (median, interquartile range, whiskers per Tukey); n = 6 per group; CG25 exhibits significantly higher stiffness than CG70 (*p* = 0.0197) and DB25 (*p* = 0.0005), and with a trend compared to pure PLA. CG70 shows the lowest stiffness within the CG series. DB25 is slightly below PLA, while DB40 and DB70 levels are comparable with or above PLA. Breaking load follows a similar pattern, with CG25 being significantly higher than DB25 (*p* = 0.0015). (**C**) Surface coverage of Trypan-Blue-stained MSCs on disk-shaped specimens at 24 h post-seeding is showing a concentration-dependent increase in cell adhesion. DB70 exhibits significantly higher surface coverage than on PLA. (**D**) Representative example of images of PLA (left), DB70 (middle), and CG70 (right) disks after 24 h post-seeding of MSCs, stained with Trypan Blue, illustrating near-confluent MSC coverage on DB70 versus sparse adhesion on PLA. Data are presented as box plots (median, IQR); *n* = 5 per group. * *p* ≤ 0.05, ** *p* ≤ 0.01, *** *p* ≤ 0.001 Statistical comparisons: Kruskal–Wallis with Dunn post hoc analysis.

The breaking load followed the same pattern, but only the comparison between CG25 and DB25 reached statistically significant differences, with CG25 exhibiting a higher breaking load (Figure 4).

### 3.3. Cell Adhesion 

Early cell adhesion mirrored the surface access of the fillers. Across all microscopy series, pure PLA consistently showed low initial cell adhesion relative to the test specimen surfaces. In contrast, PLA–DBM and PLA–CG exhibited progressively higher surface coverage with increasing filler content and time. PLA–DB already showed clear increases in MSC adhesion at 20–50% DB/CG compared to pure PLA, with high adhesion at 70%. Interestingly, there was no further visible increase between 70% and 90% CG filament, which was not further followed as it is hardly printable. Fluorescence microscopy with Calcein/DAPI was limited by green autofluorescence of the CG; therefore, it was not used for quantification (Figure 4).

**Figure 5:**
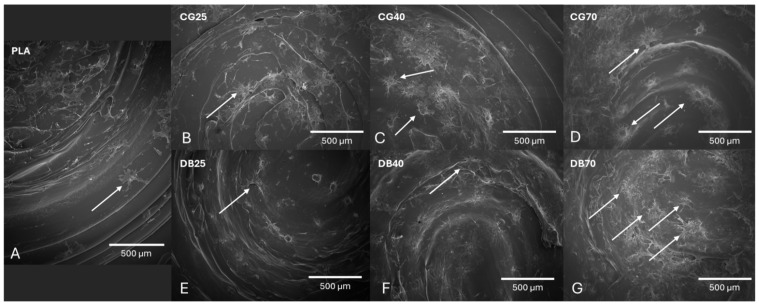
Early MSC adhesion and morphology. SEM images of MSCs cultured for 24 h on disk-shaped specimens after 24 h. (**A**) Pure PLA control shows only sparse, isolated cell attachment (white arrows) on the relatively smooth polymer surface, indicating limited initial bioactivity. (**B**–**D**) PLA–CG series (CG25, CG40, CG70). Cell density and spreading increase progressively with higher cortical granulate loading. At 40% and 70% (CG40, CG70), MSCs form denser, interconnected clusters (white arrows). (**E**–**G**) PLA–DBM series (DB25, DB40, DB70). A similar dose-dependent increase in cell adhesion is observed. DB70 exhibits the most extensive cell coverage with flattened, well-spread morphologies (white arrows) spanning the printed surface ridges. Overall, both composite types support significantly enhanced early adhesion compared to pure PLA, with the highest efficacy at 70 wt% loading.

SEM at 24 h indicated dose-dependent increases in MSC adhesion on both CG- and DBM-containing surfaces relative to PLA; cells appeared flatter and more spread at 70% filler (CG70, DB70). Thus, a SEM readout was evaluated and showed a higher surface cell density for DB70 than for CG70 (Figure 5).

### 3.4. Osteogenic Gene Expression

**Figure 6:**
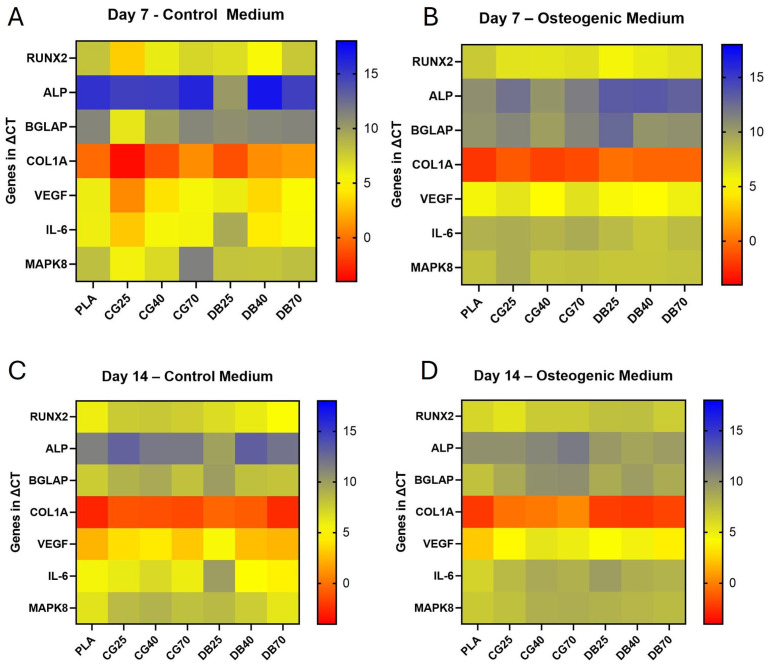
Gene expression heatmap. Heatmap visualization of normalized ΔCt values for RUNX2, ALP, BGLAP, COL1A, VEGF, IL-6, and MAPK8 in MSCs cultured on PLA, PLA–CG, and PLA–DBM disks at day 7 and day 14 under control and osteogenic (OD) media. (**A**) Day 7 control and (**B**) day 7 OD show largely similar expression patterns across materials with only subtle shifts, most notably higher expression of RUNX2 on DB40. (**C**) Day 14 control illustrates time-dependent slight increases in BGLAP on CG70 compared to day 7, indicating progression of late-stage osteogenic marker expression even without exogenous induction. (**D**) Day 14 OD reveals the most striking material-specific differences: CG40 and CG70 display distinctly lower expression of BGLAP and VEGF compared to PLA and DBM groups, confirming their significant downregulation and visualizing the delayed terminal maturation phenotype on highly mineralized CG surfaces. In contrast, DBM groups retain lower ΔCt values for COL1A and BGLAP, consistent with sustained matrix synthesis and maturation potential. IL-6 shows lower expression across most composites at day 14, particularly pronounced on DB25, reflecting the anti-inflammatory resolution. The color scale indicates relative transcript abundance (blue = higher ΔCt/lower expression; red/yellow = lower ΔCt/higher expression), enabling direct visual identification of upregulated and downregulated genes by material and condition.

**Figure 7:**
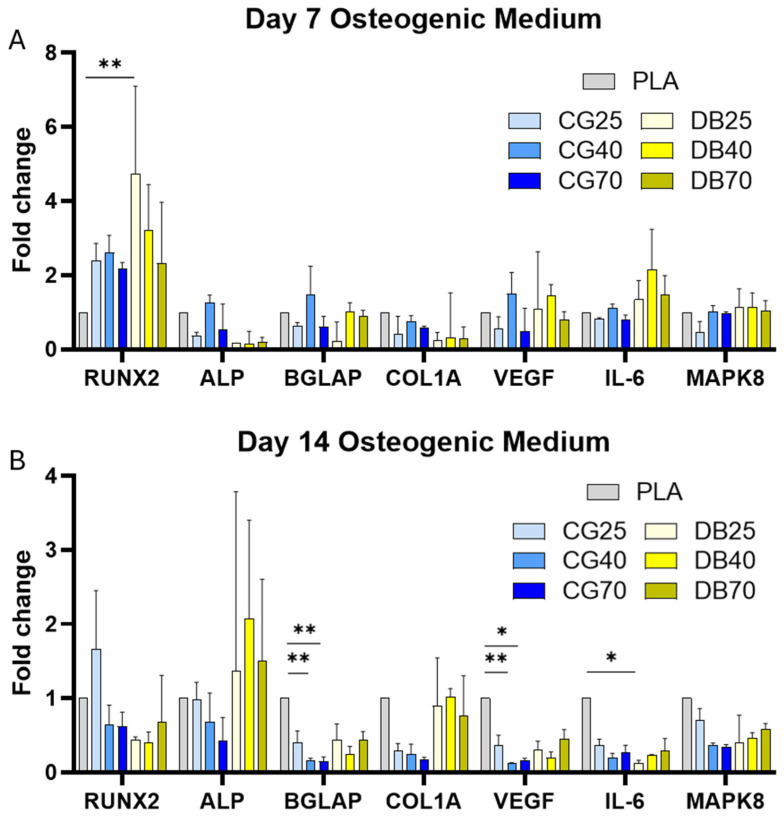
Gene expression profiles (fold change). Fold-change expression (2^−ΔΔCt, normalized to PLA control) of osteogenic, inflammatory, and pro-angiogenic markers in MSCs cultured on disk-shaped specimens under osteogenic differentiation medium. (**A**) Day 7 shows an overall modest osteogenic response with a significant early increase in RUNX2 on DB40 compared with PLA, while most other markers remain close to baseline, indicating that strong material effects on transcription have not yet fully developed. (**B**) Day 14 reveals pronounced, material-dependent expression patterns: DBM composites maintain or enhance expression of osteogenic markers (RUNX2, ALP, COL1A, BGLAP) and VEGF relative to PLA, whereas CG40 and CG70 exhibit a marked downregulation of the late osteogenic marker BGLAP (*p* = 0.0076) and the angiogenic factor VEGF (*p* ≤ 0.042), consistent with a delayed progression towards terminal osteoblast maturation on highly mineralized CG surfaces. Inflammatory signaling is attenuated at this stage, especially by a significant reduction in IL-6 on DB25 (*p* = 0.015), indicating an anti-inflammatory shift in the DBM groups. Bars represent mean ± standard deviation (SD), *n* = 3 per group; significance was tested using Kruskal–Wallis with Dunn post hoc correction (* *p* ≤ 0.05, ** *p* ≤ 0.01).

Osteogenic gene expression was profiled across seven markers (RUNX2, ALP, BGLAP, COL1A, VEGF, IL-6, MAPK8) at day 7 and day 14 under basal (control) and osteogenic differentiation (OD) media. The complete expression profile is visualized in Figure 6 (fold-change relative to PLA) and Figure 7 (heatmap of ΔCt values). 

At day 7, expression levels of osteogenic markers were generally comparable across all groups. A significant material-dependent upregulation was observed only for RUNX2 in DB40 compared to PLA (*p* = 0.009).

By day 14 under basal conditions, a temporal progression in osteogenic marker expression was evident compared to day 7. Notably, BGLAP (osteocalcin) expression increased on CG70 scaffolds over time.

Under osteogenic differentiation at day 14, distinct material-specific profiles emerged. RUNX2 and ALP showed maintained expression across groups without uniform significant differences versus PLA. However, the late-stage maturation marker BGLAP was significantly downregulated in CG40 and CG70 scaffolds compared to PLA (*p* = 0.0076; fold change < 0.2). Similarly, COL1A expression in CG groups remained lower than in PLA and DBM groups. In contrast, DBM composites (DB25, DB40) maintained BGLAP and COL1A expression levels comparable to or higher than controls, indicating a more preserved osteogenic trajectory.

Under osteogenic differentiation at day 14, distinct material-specific profiles emerged. RUNX2 expression was variable, showing an increase only in CG25 scaffolds, while remaining comparable to or lower than PLA in all other composite groups. ALP expression tended to be higher in DBM composites compared to PLA, whereas it remained reduced in CG scaffolds. Crucially, the late-stage maturation marker BGLAP was significantly downregulated in CG40 and CG70 scaffolds compared to PLA (*p* = 0.0076; fold change < 0.2). Similarly, COL1A expressions in CG groups remained lower than in PLA and DBM groups. In contrast, DBM composites maintained BGLAP and COL1A expression levels closer to controls, avoiding the sharp suppression observed in the cortical granulate series. At day 7, RUNX2 was significantly upregulated in DB40 compared to PLA (*p* = 0.009) and DB70. ALP, BGLAP, and COL1A showed no significant material-dependent differences across all groups. By day 14 under basal conditions, both BGLAP and COL1A increased significantly over time (day 7 → 14) on PLA, DB40, DB70, and CG70. RUNX2 increased modestly on PLA and DB70. ALP showed broad upregulation across both composite types.

### 3.5. Inflammatory Gene Expression

The inflammatory response (IL-6, MAPK8) showed a material-dependent modulation over time. At day 7, expression levels were low with no significant differences between materials. By day 14 under osteogenic differentiation, IL-6 expression was significantly downregulated in DB25 scaffolds compared to PLA (*p* = 0.015). A general trend of reduced IL-6 expression was observed across most composite groups relative to day 7, indicating a dampening of pro-inflammatory signaling in the differentiating cultures (Figure 6 and Figure 7).

### 3.6. Pro-Angiogenic Activity

VEGF expression was quantified to assess angiogenic signaling. At day 14 under osteogenic differentiation, VEGF expression was significantly downregulated in CG40 (*p* = 0.002) and CG70 (*p* = 0.042) scaffolds compared to PLA (fold change < 0.2). DBM composites did not show this suppression and maintained VEGF levels closer to the PLA control (Figure 6 and Figure 7).

## 4. Discussion

We established a manufacturing protocol for printable composite filaments at 25/40/70 wt% with either DBM or CG, consisting of the following steps: grinding, silicone microcoating of the feed, starved feeding, sequential (two-pass) extrusion, and post-die mixing for higher concentration or more temperature-sensitive materials. Printed specimens showed uniform filler dispersion and near-surface particle exposure on a disk-shaped scaffold, with microtopography increasing with filler load. In the three-point bending test, CG25 was stiffer than neat PLA, CG40, and CG70. DB25 is less stiff than PLA, while DB40 and DB70 showed higher stiffness. Early cell adhesion increased on both composites versus PLA. qPCR revealed nuanced, material- and time-dependent regulation across inflammatory (IL-6, MAPK8), osteogenic (RUNX2, ALP, BGLAP, COL1A), and pro-angiogenic (VEGF) markers, with osteogenic medium generally dampening inflammatory signals.

The necessity of a specialized extrusion toolkit becomes evident when considering the inherent challenges of processing irregular additives, which are prone to segregation and agglomeration. The challenges of incorporating high additive loadings (above >20 wt%) into PLA filaments without sacrificing dimensional stability and printability are a demanding task and have been widely described in different other industries where extrusion-based processing is used for combining different particle sizes in one blend [1,25,35,36]. Starved feeding increased specific energy input and promoted the deglomeration of DBM and CG within molten PLA while avoiding excessive die pressure. This approach is conceptually distinct from conventional flood-fed extrusion used in single-screw systems, where continuous barrel filling at the feed hopper can result in poor mixing efficiency and material degradation under prolonged residence times [30,37,38]. In contrast, the metered starve-fed approach allows the screw to operate at controlled under-filled conditions, enabling higher shear stresses in the intermeshing zone while maintaining lower overall temperatures—a critical advantage when processing thermally sensitive bioactive components.

At very high filler loadings, a sequential masterbatch-palletization-dilution pass disrupted weak filler–filler contacts formed during cooling and improved dispersion. This two-pass strategy mirrors industrial approaches used successfully for glass-fiber and mineral-filled thermoplastics and has been shown to yield more uniform concentration profiles and reduced agglomeration compared with single-pass extrusion [39].

Post-die mixing further attenuated concentration fluctuations prior to sizing and spooling, addressing the common issue of “surging” or oscillatory composition waves that can arise during transitions between extrusion zones [38].

Together with silicone-assisted pre-coating, these measures enabled stable spooling at the target diameter and reliably printing across all filler loadings. 

The silicone microcoating step is particularly noteworthy. In practice, it yielded visibly more homogeneous blends at low absolute filler masses and stabilized dosing into the extruder across all target loadings by reducing inter-particle friction, minimizing dusting or static force, and suppressing segregation during the blend preparation. The silicone quantity was deliberately kept minimal to avoid plasticization or flow alteration downstream. It facilitated reproducible dosing even at very small batch sizes (10 g in the present study), a constraint imposed by the limited absolute masses of additives required for high wt% formulations [38,40]. Such small batch sizes are particularly beneficial for medical and in vitro research, where only limited material quantities are available, and added substances are required. While silicone loading was intentionally kept minimal to avoid plasticization or altered flow behaviors, this pre-coating strategy provides a practical solution to the scalability challenge in laboratory-scale filament manufacturing involving hydrophobic polymer carriers and polar bioactive additives.

Prior to establishing the optimized protocol, processing high-filler-loading composites proved challenging. Initial extrusion attempts—performed without the established protocol—yielded filaments characterized by significant dimensional instability and macroscopic inhomogeneities (Figure 2). These defects manifested as local particle agglomerations and diameter fluctuations (“diameter jumps”) exceeding the tolerance for reliable FFF. Notably, processability deteriorated sharply with rising filler content; while 25 wt% loading remained marginally extrudable, higher filler loadings frequently led to filament breakage during spooling or unprintability, risking nozzle obstruction. The introduction of the specific manufacturing toolkit was therefore critical to suppress this instability, and using this feed strategy, PLA composite filaments containing CG or DBM with up to 90%, which then, at this high dosage, is barely printable. were produced at 25%, 40% and 70% (wt/%) filler and could be spooled at a controlled diameter suitable for fused-filament fabrication. 

The resulting filament microstructure and surface characteristics were central to the enhanced cellular response observed in our experiments. SEM of filament cross-sections and printed disk-shaped specimens showed uniform distribution of CG and DBM throughout the PLA matrix at each concentration. SEM and microscopic panels at 24 h consistently showed greater cell coverage on composites than on PLA, with flatter/spread morphologies at higher loads (CG70, DB70). The Trypan Blue assay indicated DB70 > CG70 at 24 h. These observations are consistent with the mechanisms proposed in the following:

On the test specimens, particles were exposed at the surface in a concentration-dependent fashion: partially uncovered inclusions produce localized microtopographic features with lateral dimensions on the order of ~5–10 μm (width) and up to ~50 μm (length). Surface unevenness increased with filler fraction and was comparable in form and scale between CG- and DBM-containing materials. These features plausibly contribute to the higher early adhesion seen on composites relative to PLA in this and in the literature [41,42].

The surface microtopography revealed in this study aligns with the well-established principle of contact guidance, whereby cells align and elongate along surface features. Nanoscale grooves and ridge structures have been shown to profoundly modulate osteoblast adhesion, spreading, and gene expression. In particular, groove topographies on the order of 10 μm width induce polarized, elongated cell morphology and direct focal adhesion orientation relative to the groove direction, a phenomenon demonstrated in primary human osteoblasts cultured on photolithographically patterned substrates. The microtopographic features generated by partially exposed filler particles in the present work fall precisely within the range known to elicit contact guidance responses [41,43]. A quantitative assessment of surface topography, for instance via atomic force microscopy (AFM), was not performed. However, the significantly enhanced early MSC adhesion on the composite scaffolds compared to the smooth, bioinert PLA surfaces strongly suggests that these structural irregularities, combined with the exposure of the biological filler, effectively increased the effective surface area and provided more favorable cues for cellular attachment. Future studies should include quantitative roughness parameters to further correlate specific topographic profiles with long-term osteogenic differentiation.

More fundamentally, these topographic cues engage integrin-mediated adhesion mechanisms that underpin both cell attachment and mechanotransduction. Integrin heterodimers (particularly α5β1 and αvβ3) recognize arginine–glycine–aspartic acid (RGD) motifs present in bone matrix proteins such as fibronectin, collagen type I, and osteopontin [44]. Upon ligand binding, integrin clustering and the recruitment of focal adhesion proteins (including vinculin, talin, paxillin) lead to the formation of adhesion complexes that serve as both mechanical anchors and biochemical signaling nodes. The assembly of these focal adhesions depends critically on inter-ligand spacing (reported to be optimal between <60–140 nm) and the dimensional accessibility of matrix features at the cell–substrate interface [45,46,47]. Consequently, the exposed mineral and protein-rich filler particles within the composite filaments thus provide a more complex and dimensionally heterogeneous adhesion substrate compared to the featureless PLA surface, thereby facilitating integrin clustering and downstream mechanotransduction signaling associated with focal adhesion maturation.

PLA is an intrinsically hydrophobic synthetic aliphatic polyester with reported water contact angles between 75 and 85°, which represents a significant barrier to cell adhesion and tissue integration. This hydrophobicity limits the adsorption of cell-adhesive proteins (such as fibronectin, vitronectin, and collagen) from serum and culture media onto the material surface [47,48,49,50,51]. In contrast, more hydrophilic surfaces with water contact angles below 65° preferentially promote the selective adsorption of cell-adhesive proteins [52] over immune-activating opsonins, thereby favoring anti-inflammatory responses and enhanced osteoblast recruitment [47,53].

While not directly measured in this study, the incorporation of protein-rich DBM and mineralized CG is suggested to alter the surface wettability of the predominantly hydrophobic PLA matrix. Based on the chemical composition of these biological fillers, it is hypothesized that the presence of hydrophilic amino acid sequences and mineral sites provides a more favorable environment for initial protein adsorption, which in turn could facilitate the observed MSC attachment. When partially exposed at the composite surface, HA and calcium phosphate particles create localized hydrophilic domains that increase the effective surface hydrophilicity of the composite relative to pure PLA [47,54,55]. Future studies utilizing modified water contact angle measurements are needed to precisely quantify these surface energy changes.

The partially exposed mineral and protein phases on the PLA–DBM and PLA–CG composite surfaces directly mitigate this hydrophobic limitation. Hydroxyapatite (HA) and calcium phosphate minerals, the dominant inorganic phases of CG, are intrinsically hydrophilic, with surface hydroxyl (-OH) and phosphate (PO_4_^3−^) groups that form hydrogen bonds with water molecules. These mineral-rich surfaces achieve rapid water uptake and wettability. 

This interpretation is consistent with our findings of improved early MSC attachment on the composite filaments. The significantly higher cell-covered surface area observed for DB70 versus CG70 at 24 h can, at least in part, be attributed to enhanced surface hydrophilicity. DBM contains a substantial organic matrix of collagen type I proteoglycans and residual growth factors that further enhance surface hydrophilicity compared with purely mineral CG. Exposed collagen fibrils at the DBM composite surface are likely to create a biomimetic extracellular matrix-like microenvironment superior to pure PLA, which underlines the superior early cell adhesion. 

The dependence of hydrophilicity on filler loading is also noteworthy. As CG and DBM filler content increases from 25% to 70 wt%, the surface density of exposed mineral and protein phases increases, progressively reducing effective overall water contact of the composite. This trend provides a mechanistic explanation for the concentration-dependent enhancement of cell adhesion and spreading observed in our SEN assessment: higher filler loadings yield more hydrophilic surfaces that promote more robust integrin-mediated adhesion and downstream mechanotransduction.

From a translational perspective, enhancement of the surface properties is functionally critical during early in vivo implantation. Early scaffold integration (day 0–7) is dominated by protein-mediated adhesion events. Hydrophilic mineral surfaces exhibit increased surface free energy (40–55 mJ/m^2^ for polarized HA and calcium phosphate, compared to ~35 mJ/m^2^ for PLA), which favors adsorption of bone-relevant extracellular matrix proteins from serum, including fibronectin, vitronectin, type I collagen, and bone sialoprotein. [56]. These adsorbed proteins present their RGD sequences and other integrin-binding motifs in accessible conformations, enabling rapid integrin-ligand engagement [44,46,51]. 

As stated by Monopoli et al. (2011), a protein corona forms on scaffold surfaces within minutes of contact with biological fluids, critically determining downstream cellular responses [57]. The surface modification by DBM or CG might also change the protein corona building up around the scaffold compared to pure PLA. Moreover, the hydrophilic mineral surfaces facilitate the formation of a stable aqueous layer at the material–cell interface, which is essential for integrin-ligand diffusion and clustering into focal adhesion complexes. In contrast, hydrophobic surfaces disrupt this aqueous microenvironment, reducing integrin mobility and impairing mechanotransduction signaling [44,46].

Regarding the mechanical integrity of the constructs, the bending response was found to be a sensitive function of both filler identity and loading. CG25 showed increased stiffness relative to neat PLA and to higher CG contents, while CG70 exhibited reduced stiffness compared with CG40. Conversely, DB25 resulted in a modest decrease in stiffness relative to PLA, whereas DB40 and DB70 climbed to levels comparable to PLA and significantly above DB25. A direct comparison at 25 wt% further highlighted that CG25 was substantially stiffer than DB25. A reasonable interpretation is that moderate mineral loading (CG25) reinforces the PLA matrix, whereas very high filler fractions introduce stress concentrators and interfacial defects that limit stiffness and ultimate load. In the case of DBM, the protein-rich phase may act as a relatively compliant inclusion at low loadings, while improved load transfer at higher filler fractions likely underlies the stiffness recovery observed at DB40 and DB70.

This composition-dependent mechanical behavior reflects two competing structural phenomena. First, the inorganic mineral phase of CG, predominantly HA, possesses a Young’s modulus of 40–120 GPa, far exceeding that of PLA (~2–4 GPa) [51,58]. Accordingly, at moderate filler loadings, particulate CG acts as a stiff reinforcement, increasing composite stiffness in accordance with classical composite theory. However, at very high loadings (CG70), the effective spacing between particles diminishes, generating stress concentration sites at particle–matrix interfaces and within agglomerated clusters. 

In contrast, the organic, protein-rich component of DBM behaves as a soft, somewhat viscoelastic component, which at low loadings may act as a plasticizer or stress concentrator, reducing matrix stiffness. As DBM content increases, the absolute mineral content also rises, and the improved percolation of the mineral phase through the PLA matrix promotes load transfer and composite stiffening. 

From a translation perspective, the mechanical performance for resorbable bone scaffolds is narrow: constructs must provide sufficient stiffness and strength to prevent scaffold and bioreactor collapse during the implantation and critical early healing phase, yet degrade at a rate synchronized with new bone deposition. Therefore, the ability to tune bending behavior through filler identity and loading represents a critical design parameter for FFF-fabricated, bioactive composite scaffolds.

Moving to the molecular level, the expression of osteogenic markers revealed material- and time-specific regulation patterns. The CG series (particularly CG40 and CG70) demonstrated a specific expression pattern under osteogenic conditions, with maintenance of early markers (RUNX2, ALP) but significant downregulation of the late maturation marker BGLAP and the angiogenic factor VEGF. This suggests that cells on the high-stiffness, purely mineral CG surfaces may be retained in an early proliferative or committed state, with a delay in progression to terminal mineralization compared to PLA. The suppression of VEGF in these groups mirrors the BGLAP reduction and may reflect a metabolically quiescent phenotype with reduced paracrine signaling requirements at this specific time point. This kinetic delay is likely driven by the lack of organic signaling motifs (collagen, growth factors) on the mineral surface, forcing differentiation to proceed primarily via mechanotransduction, which can alter the timing of phase transitions.

In contrast, DBM composites demonstrated a more balanced trajectory. DBM groups maintained expression of both early and late markers (BGLAP, COL1A) as well as VEGF at day 14, without the suppression observed in CG. The early elevation of osteogenetic markers on DBM shows its intrinsic osteoinductive capacity through residual growth factors (BMPs, TGF-β), as also seen in our previous work [25,59]. These intrinsic osteoinductive proteins and collagen type I in the demineralized matrix provide biochemical cues that facilitate a complete and synchronized differentiation cascade, demonstrating synergy between material properties and differentiation factors [60]. 

VEGF regulates angiogenesis and osteogenic commitment of progenitors in a dose-dependent manner. Critically, osteo-angiogenic coupling requires a narrow VEGF concentration range; moderate early VEGF supports vascular invasion, while excessive levels paradoxically impair bone formation through osteoclast recruitment and delayed vascular integration [60,61,62]. 

In our study, VEGF expression demonstrated a material-independent, time-dependent pattern. At day 7 under both control and osteogenic differentiation media, VEGF remained at moderate, relatively consistent levels across all composite types, indicating that the scaffold material composition did not dramatically alter pro-angiogenic signaling at this early time point. By day 14 under osteogenic differentiation conditions, VEGF expression showed a subtle but systematic attenuation across all groups, consistent with the known suppression of VEGF during osteogenic commitment. This material-independent maintenance of moderate VEGF levels—rather than excessive elevation or severe suppression—reflects a physiologically balanced phenotype. The stable VEGF expression across both early and differentiation phases allows for simultaneous vascular signaling and osteogenic commitment, supporting effective osteo-angiogenic coupling without the destabilizing effects of excessive pro-angiogenic drive that would otherwise impair bone formation [62].

In terms of the inflammatory context, the transient activation of markers such as IL-6 and MAPK8 suggests a physiological response to material exposure rather than chronic inflammation. At day 7 under basal conditions, IL-6 was elevated on DB40 and DB70 and moderately increased on CG25 compared to lower-loading composites. This early elevation represents the initial innate immune activation triggered by material exposure, including integrin ligation, topographic stimulation, and potential release of damage-associated molecular patterns (DAMPs). Critically, this early, moderate IL-6 elevation is not inherently pathological; in the bone tissue engineering context, IL-6 at physiological levels supports MSC proliferation through ERK1/2 signaling and can facilitate early scaffold colonization and matrix deposition. The pattern observed, with loading-dependent IL-6 increases at day 7, is consistent with a dose-responsive material stimulus rather than chronic inflammation [63,64].

By day 14 under osteogenic differentiation conditions, a marked attenuation of IL-6 was observed across most groups, with a significant reduction in DB25. This likely reflects the known mechanism of osteogenic differentiation and the resolution of the material-induced inflammatory phase. Exposure to glucocorticoids, inorganic phosphate, and ascorbic acid triggers a molecular switch wherein the IL-6 signaling axis shifts from a proliferation-promoting context toward a differentiation-promoting context in which IL-6 levels are suppressed, and p38 MAPK, SMAD, and Wnt/β-catenin signaling dominate. This temporal pattern mirrors the physiological immune response during fracture healing, where an initial inflammatory phase (days 0–3 post-injury) with elevated IL-6, TNF-α, and IL-1β gives way to a resolution phase (days 7–14) marked by reduced pro-inflammatory signaling and emergence of pro-regenerative mediators (IL-10, TGF-β) [63,64,65].

MAPK8 (c-Jun N-terminal kinase, JNK) showed minimal elevation at day 7 and generally remained low or decreased at day 14 across most groups. This low JNK activity is favorable, as JNK is primarily associated with stress responses and pro-inflammatory signaling. The absence of JNK activation indicates that cells are not experiencing chronic oxidative stress or inflammatory stimulus from the materials, further supporting the biocompatibility profile. 

The general dampening of both IL-6 and MAPK8 under osteogenic conditions indicates that the pro-differentiation signals (dexamethasone, β-glycerophosphate) suppress inflammatory/stress-response pathways in favor of osteoblastic transcription factors, which favors successful osteogenic commitment [36,44,66,67].

When evaluating the overall performance of these composite systems relative to pure PLA, their properties can be effectively contextualized within the ‘diamond concept’ of bone regeneration. Relative to pure PLA, both composite systems improved early MSC adhesion and modulated osteogenic gene expression while remaining compatible with fused filament fabrication. PLA–DBM leverages biochemical signaling derived from its protein-rich organic phase with microtopographic cues to promote early osteogenic commitment, as reflected by increased RUNX2 and ALP expression. In contrast, PLA–CG primarily provides an osteoconductive and mechanically reinforcing mineral surface at moderate loadings (CG25), with reduced mechanical performance observed at the highest filler contents.

The diamond concept of bone regeneration, originally articulated by Giannoudis et al. in 2007 [4], describes essential requirements for effective bone repair: osteoconductivity, osteoinductivity, osteogenic cells, and mechanical stability, with vascularization acting as a critical integrative component. The performance of the PLA–DBM and PLA–CG composites can be contextualized within this framework as follows.

Osteoconductivity: Both PLA–DBM and PLA–CG provide robust osteoconductive properties. The mineral-rich composite surface (mainly for CG-containing filaments) mimics native bone mineral and supports cell adhesion, migration, and spreading within 24 h. In addition, microtopographic features in the 5–50 μm range facilitate integrin clustering and mechanotransduction, promoting early cell–material interactions.

Osteoinductivity: PLA–DBM demonstrates clear osteoinductive potential by providing matrix-bound growth factors, including BMPs, TGF-β, VEGF, and IGF-1 within its organic phase. In contrast, PLA–CG is osteoconductive but not intrinsically osteoinductive; however, when combined with exogenous osteogenic signals, CG-containing scaffolds can positively influence osteogenic differentiation.

Osteogenic cells: Both composite systems support MSC adhesion, spreading, and at least influence the differentiation process. The presence of degradation products (particularly from DBM) and the mechanical properties of the scaffold are likely to create a microenvironment favorable for MSC recruitment and lineage commitment.

Vascularization: Early VEGF expression and the sustained VEGF expression on PLA and DB70 suggest that these composites may promote pro-angiogenic signaling. However, the attenuation of VEGF under osteogenic conditions indicates that the scaffold does not generate pathologically excessive angiogenic signals—a favorable balance for synchronized osteo-angiogenic coupling.

Mechanical stability: The classical view of bone regeneration emphasizes that mechanical stability must be maintained during the critical healing window (weeks 1–8) to prevent scaffold collapse from the surrounding tissue pressure. The DBM-PLA and CG-PLA scaffolds in a future clinical application should be able to resist that pressure, whereas the critical loads, e.g., during walking, must be borne by further fixation. 

Thus, especially PLA–DBM represents a material that addresses all diamond elements (excluding host-derived biological factors not captured in an in vitro model) through its combination of scaffold architecture (FFF-printed porosity, microtopography), intrinsic osteoinductivity, support for osteogenic cell functions, and balanced pro-angiogenic signaling.

In conclusion, taking the manufacturing process as well as the biological assays into account, the results demonstrate that the mechanism-guided toolkit is effective up to a 70 wt% loading, though 40 wt% represents the ‘sweet spot’ for maintaining ideal filament diameter consistency and surface quality. DBM40 emerged as the superior composition for triggering early osteogenic signaling (e.g., RUNX2 expression) and promoting robust cellular attachment, making it the preferred choice for applications requiring high bioactivity and maintaining processability.

Despite these promising results, several methodological limitations must be acknowledged. This study employed an in vitro model using pooled human bone marrow-derived MSCs, which does not fully recapitulate the complex multicellular environment of in vivo bone regeneration, including host inflammatory responses, endothelial cell infiltration, or osteoclast-mediated resorption and remodeling. Thus, the logical next step is to prepare an animal project with a critical-size defect to evaluate all of those essential pillars for bone regeneration. However, it was primarily demonstrated that the material properties of the scaffolds presented here have positive properties in the in vitro model regarding bone healing. 

Several methodological limitations should be noted. First, the sample size for gene expression analysis was modest (*n* = 3 per group), which, while sufficient for initial characterization, limits the statistical power for detecting small effect sizes and warrants validation in larger cohorts. Second, quantification of cell adhesion was complicated by the intrinsic autofluorescence of the CG phase, which precluded reliable fluorescence-based morphometry.

With this work, we introduce our manufacturing protocol with starved feeding, sequential passes, post-die homogenization, and a simple silicone pre-coating, enabling reliably printable bioactive filaments. Filler identity and loading can be tuned to the intended biological window: the transition from in vitro to preclinical in vivo studies is a critical next step. A rat femoral critical-size defect model would allow assessment of (1) scaffold degradation kinetics and integration with host bone, (2) inflammatory response and resorption by osteoclasts/macrophages, (3) real vascularization and angiogenic bridging, and (4) de novo bone formation and mechanical restoration. 

## Data Availability

The data supporting the findings of this study are available from the corresponding author upon reasonable request.

## Abbreviations

The following abbreviations are used in this manuscript:

FFF	Fused Filament Fabrication
CSD	Critical-Size Bone Defect
PLA	Polylactic Acid
DBM	Demineralized Bone Matrix
CG	Cortical Granulate
MSC	Mesenchymal Stromal Cells
OD	Osteogenic Differentiation
SEM	Scanning Electron Microscopy
qPCR	Quantitative Polymerase Chain Reaction
Ct	Cycle Threshold
ΔCt/ΔΔCt	Delta Ct/Delta-Delta Ct
RUNX2	Runt-Related Transcription Factor 2
ALP	Alkaline Phosphatase
BGLAP	Bone Gamma-Carboxyglutamate Protein (Osteocalcin)
COL1A	Collagen Type I Alpha
VEGF	Vascular Endothelial Growth Factor
IL-6	Interleukin 6
MAPK8	Mitogen-Activated Protein Kinase 8
PBS	Phosphate-Buffered Saline
FCS	Fetal Calf Serum
DMEM	Dulbecco’s Modified Eagle Medium
P/S	Penicillin/Streptomycin
CFSE	Carboxyfluorescein Diacetate Succinimidyl Ester
DMSO	Dimethyl Sulfoxide
HMDS	Hexamethyldisilazane
HA	Hydroxyapatite
BMP	Bone Morphogenetic Protein
TGF-β	Transforming Growth Factor Beta
FAK	Focal Adhesion Kinase
ERK1/2	Extracellular Signal-Regulated Kinases 1/2
JNK	c-Jun N-Terminal Kinase
HIF-1α	Hypoxia-Inducible Factor 1-Alpha
ECM	Extracellular Matrix
